# Supplementary material for: Improvement of the Solubilization and Extraction of Curcumin in an Edible Ternary Solvent Mixture
Source: Molecules. 2021 Dec 20;26(24):7702. doi: 10.3390/molecules26247702 (PMC8703436; doi:10.3390/molecules26247702)
Supplement: Supplementary file 1 [file molecules-26-07702-s001.zip › molecules-1492934-supplementary.pdf]

Electronic Supplementary Material

Improvement of the Solubilization and Extraction of Curcumin in an Edible Ternary Solvent Mixture

Verena Huber*, Johnny Hioe, Laurie Muller, Pierre Degot, Didier Touraud, and Werner Kunz*

Institute of Physical and Theoretical Chemistry, University of Regensburg, D-93040 Regensburg, Germany

* Correspondence: verena1.huber@ur.de (V.H.); werner.kunz@chemie.uni-regensburg.de (W.K.)

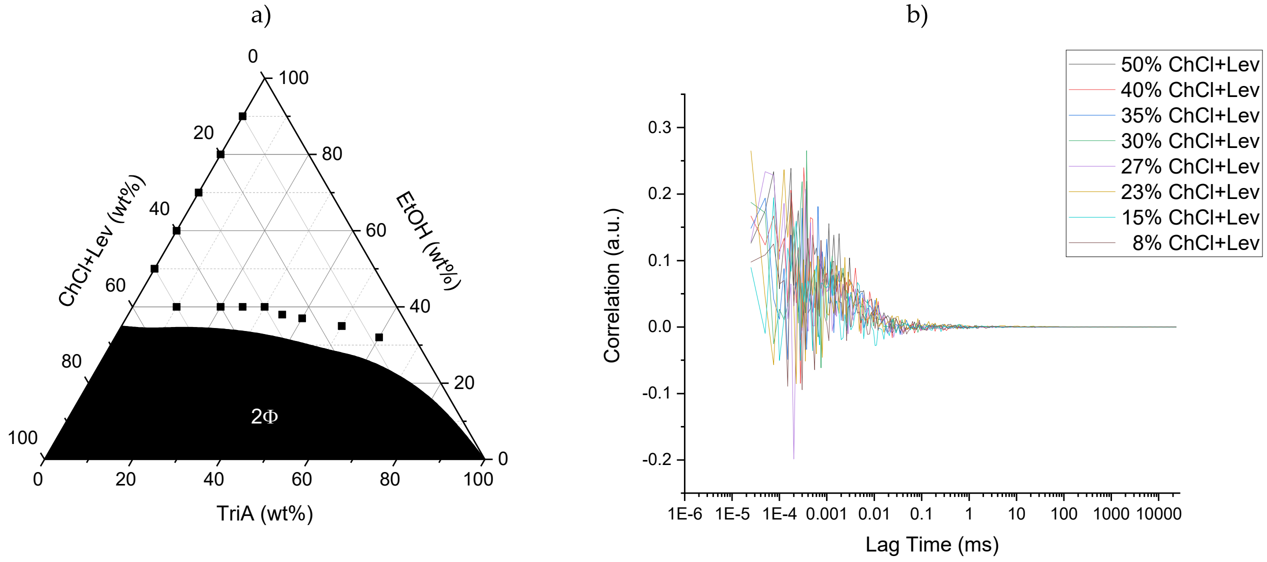

Figure S1: a) Ternary phase diagram of ChCl+Lev/EtOH/TriA with the ternary and binary points examined via DLS and b) correlation curves in the ternary system ChCl+Lev/EtOH/TriA close above the two-phasic region.

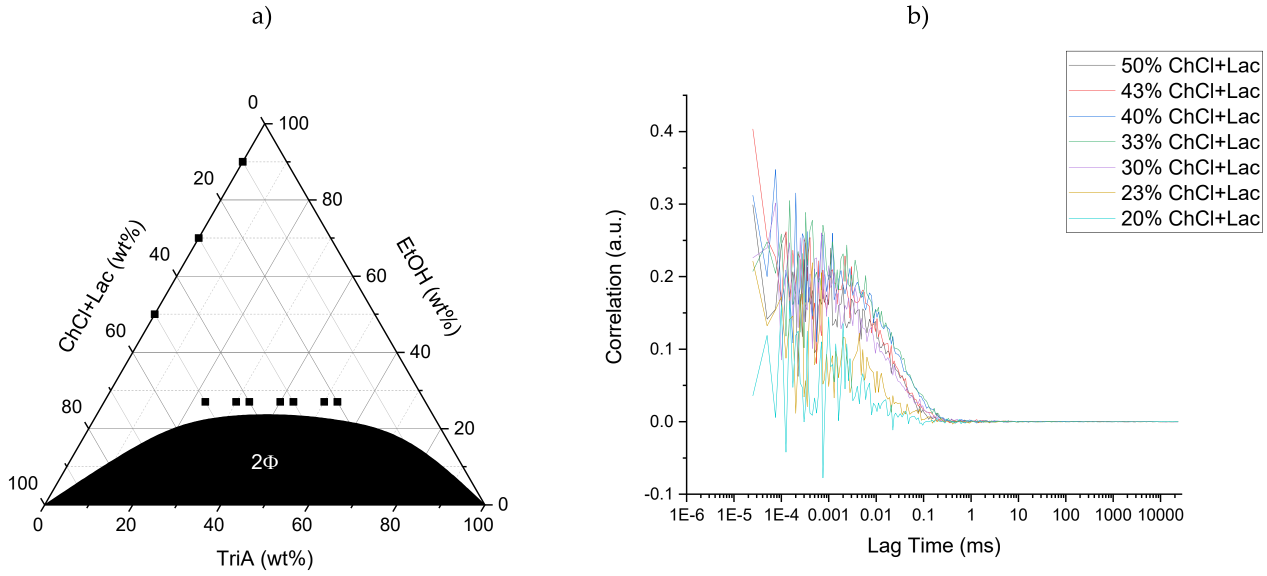

Figure S2: a) Ternary phase diagram of ChCl+Lac/EtOH/TriA with the ternary and binary points examined via DLS and b) correlation curves in the ternary system ChCl+Lac/EtOH/TriA close above the two-phasic region.

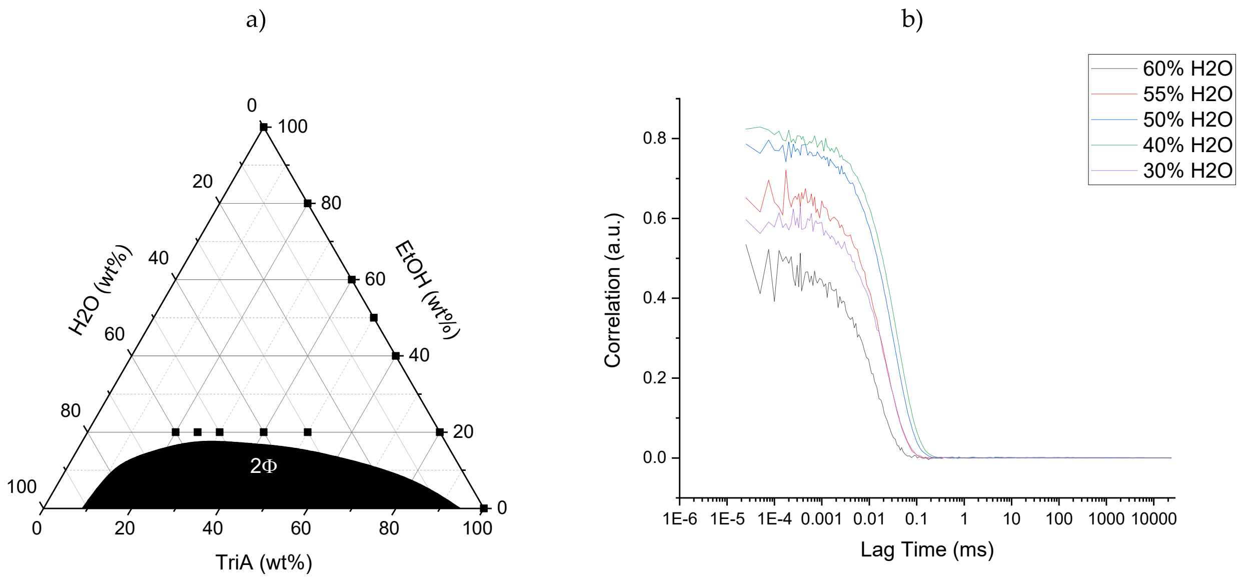

Figure S3: a) Ternary phase diagram of H_2O/EtOH/TriA with the ternary and binary points examined via DLS and b) correlation curves in the ternary system H_2O/EtOH/TriA close above the two-phasic region.

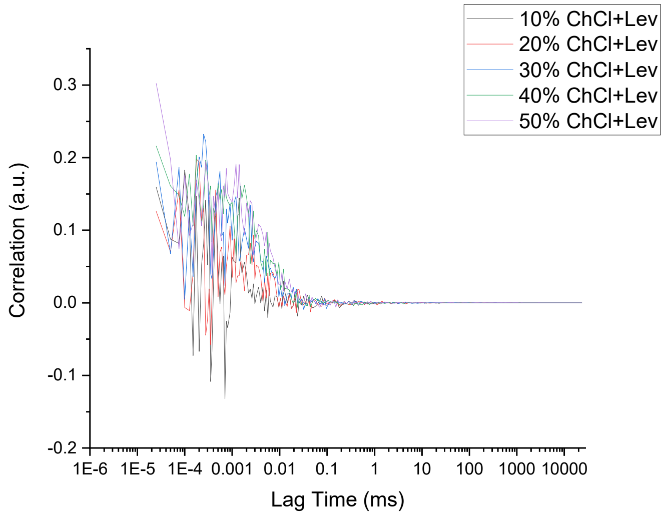

Figure S4: Correlation curves in the binary system ChCl+Lev/EtOH. The respective points can be viewed in Fig. S 1 a.

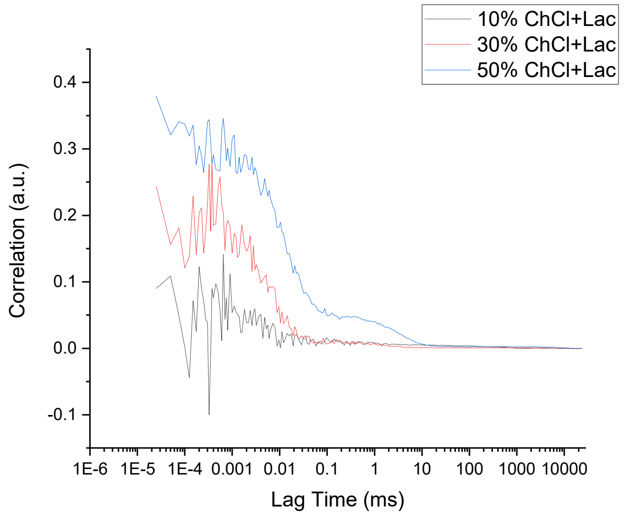

Figure S5: Correlation curves in the binary system ChCl+Lac/EtOH. The respective points can be viewed in Fig. S 2 a.

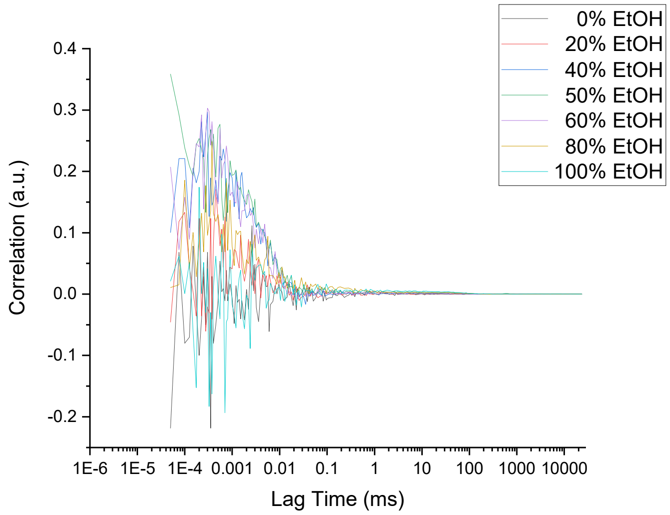

Figure S6: Correlation curves in the binary system EtOH/TriA. The respective points can be viewed in Fig. S 3 a. The data were provided by P. Degot et *al.* [3].

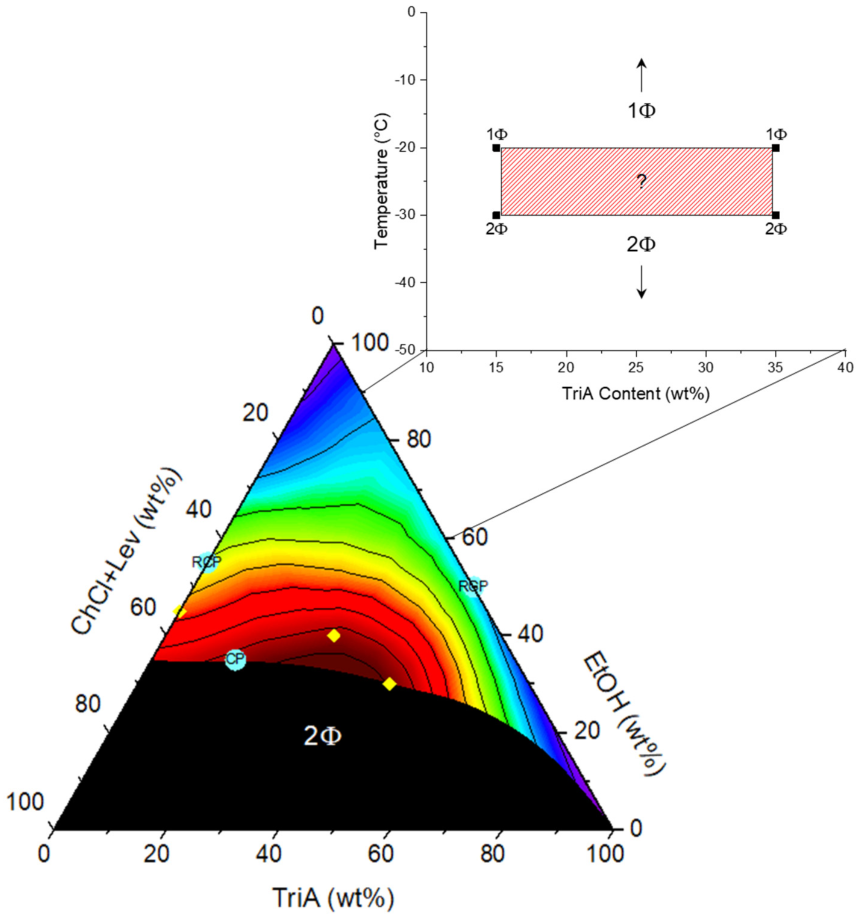

Figure S7: Binary, temperature-dependent phase diagram of triacetin and ethanol with liquid-liquid equilibrium points at -20 °C and -30 °C in context with the ternary phase diagram at 25 °C. At -20 °C no LLE point was found, whereas at -30 °C it was. The hatched area in the binary phase diagram at the top is an indication of the area of uncertainty in which the phase transition should happen.

Generally, the vapor pressure of a pure liquid compound p_i^0 at temperature T is required for the calculation of phase diagrams. If p_i^0 is not experimentally measured at the desired temperature, for example at -30.0 °C for TriA, then it must be estimated by either the Antoine equation (see section 3.2.4, Eq. 1) or the Wagner equation (see section 3.2.4, Eq. 2). For the fitting of Wagner coefficients at least 6 experimental data points must be available, while for the fitting of Antoine coefficients only 3 experimental data points are required.

As a matter of fact, the closer the temperature of the experimental data points is to the desired temperature, the better is the fitting of the coefficients and, therefore, the better is the estimated p_i^0 at the desired temperature. The availability of the experimentally determined vapor pressure of ethanol over a wide temperature range (-31.3 °C to +242 °C) allows better estimation for the vapor pressure at low temperature (-40.0 °C). The extrapolated vapor pressure of ethanol p_{EtOH}^0 at -40 °C with the respective Antoine/Wagner coefficients equals 1.508 mbar according to the Antoine equation or respectively 1.514 mbar according to the Wagner equation, and thus are very similar. In contrast, for triacetin, experimental data points were available ranging only from 11.09 °C to 45.05 °C [30]. Thus, the derived Antoine's coefficients from the Antoine's vapor pressure equation (Eq1) for triacetin equal $\boldsymbol{A}$ = 27.3021, $\boldsymbol{B}$ = 9964.0846, and $\boldsymbol{C}$ = 1.4214. With the derived coefficients, the extrapolated vapor pressure of pure triacetin p_{TriA}^0 at -40.0 °C K was estimated to be 1.457 x 10^{-6} mbar. Since the vapor mole fraction y_i is obtained from the ratio of partial

pressure $p_i^0 x_i \gamma_i$ and total vapor pressure p_{tot} (see section 3.2.4, Eq 3.), it implies that y_i at low temperatures is also lowered when the vapor pressure of the pure liquid compound p_i^0 is significantly low. The attenuation of y_i by the activity coefficient γ_i and mole fraction x_i becomes negligible. This is particularly true in the case of triacetin. The condition for an LLE between two liquid phases $'$ and $''$ requires $x_i' \gamma_i' = x_i'' \gamma_i''$ for all compounds i and thus, the calculation of LLE-points was solved iteratively. Since the vapor pressures of the two pure compounds differed significantly, the condition for LLE was barely fulfilled. In other words, the convergence in the iterative procedure to find LLE points was hardly achieved.

Table S1: Gradient HPLC procedure for the analysis of the three curcuminoids after P. Degot et *al.* [3].

Time (min)	Solution A (%)	Solution B (%)
0-17	60-40	40-60
17-18	40-0	60-100
18-24	0	100
24-25	0-60	100-40
25-32	60	40

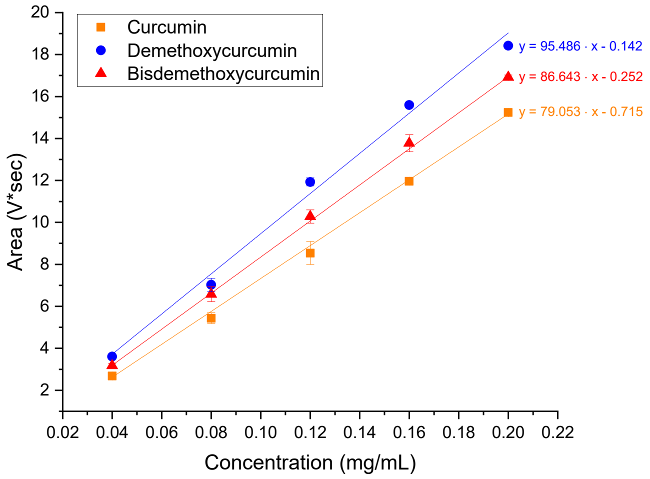

Figure S8: Calibration curves of curcumin (orange square), demethoxycurcumin (blue circle), and bisdemethoxycurcumin (red triangle) obtained by elution via HPLC.

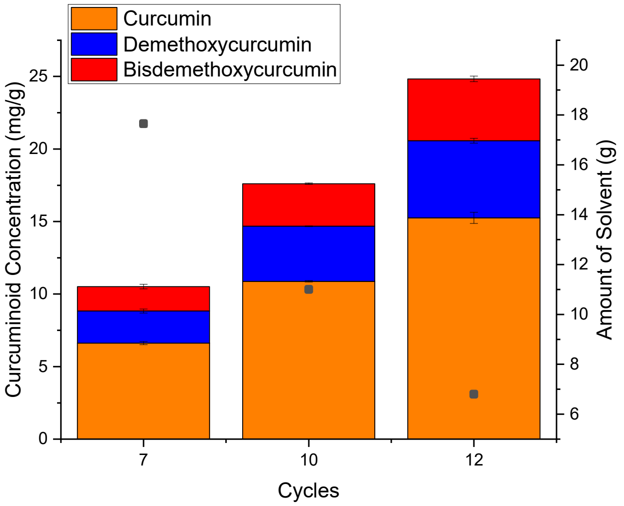

Figure S9: Curcuminoid concentration in mg/g solvent after 7, 10, and 12 extraction cycles. The gray squares represent the amount of solvent remaining after every cycle.

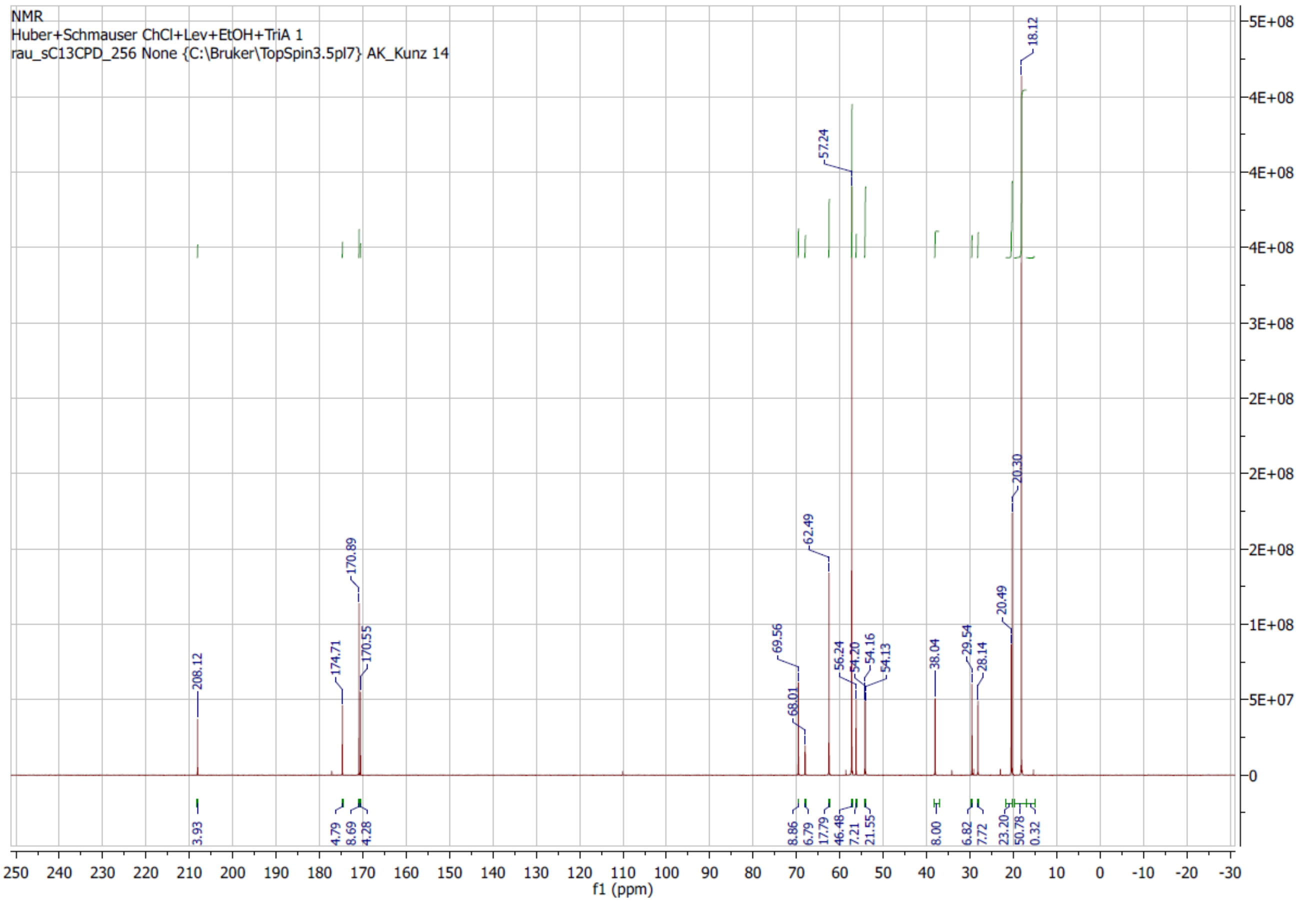

Figure S10: ^{13}C-NMR spectrum of the ternary extraction solvent of ChCl+Lev/EtOH/TriA (30:40:30) in weight fresh after preparation.

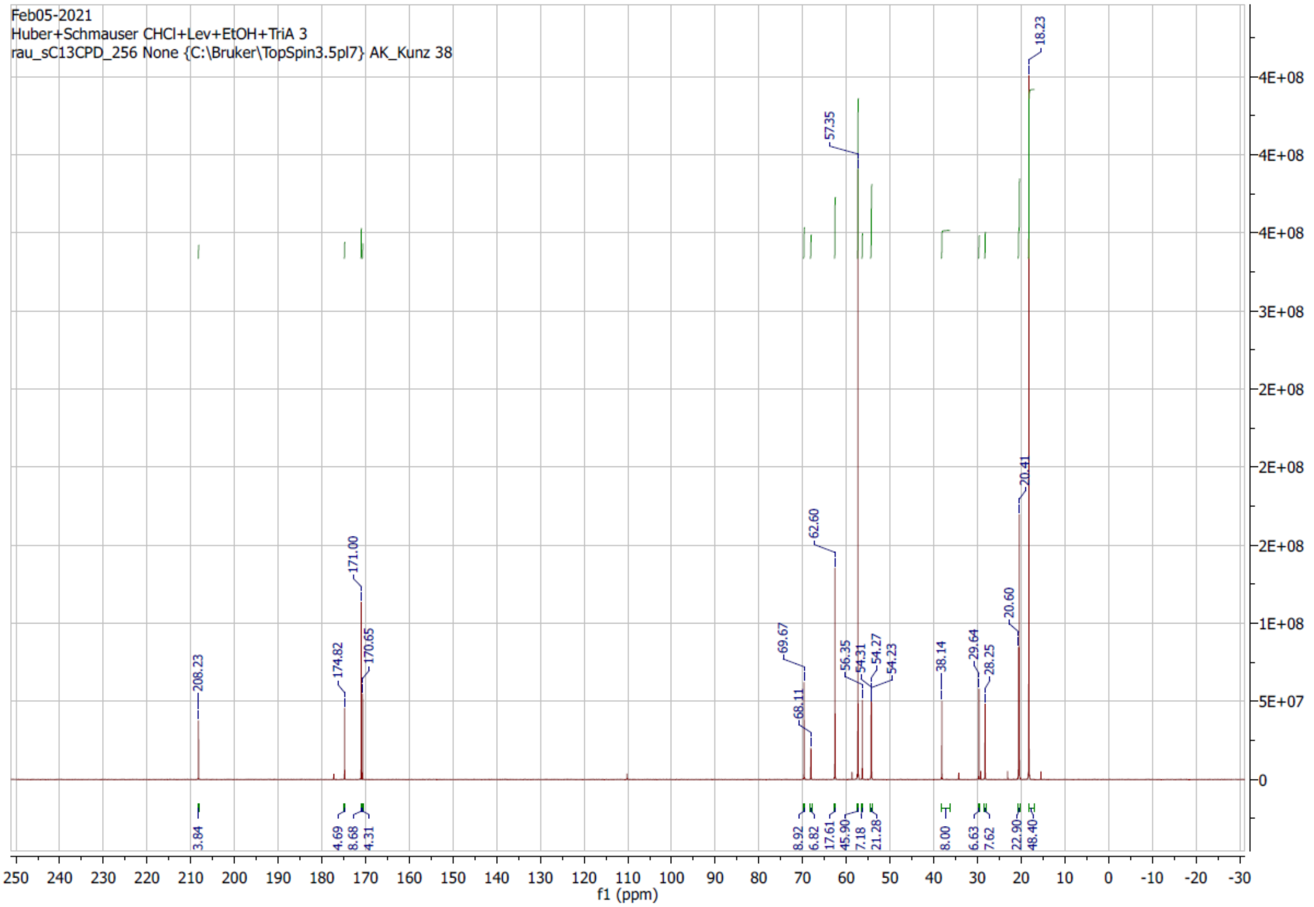

Figure S11: ^{13}C-NMR spectrum of the ternary extraction solvent of ChCl+Lev/EtOH/TriA (30:40:30) in weight one day after preparation.

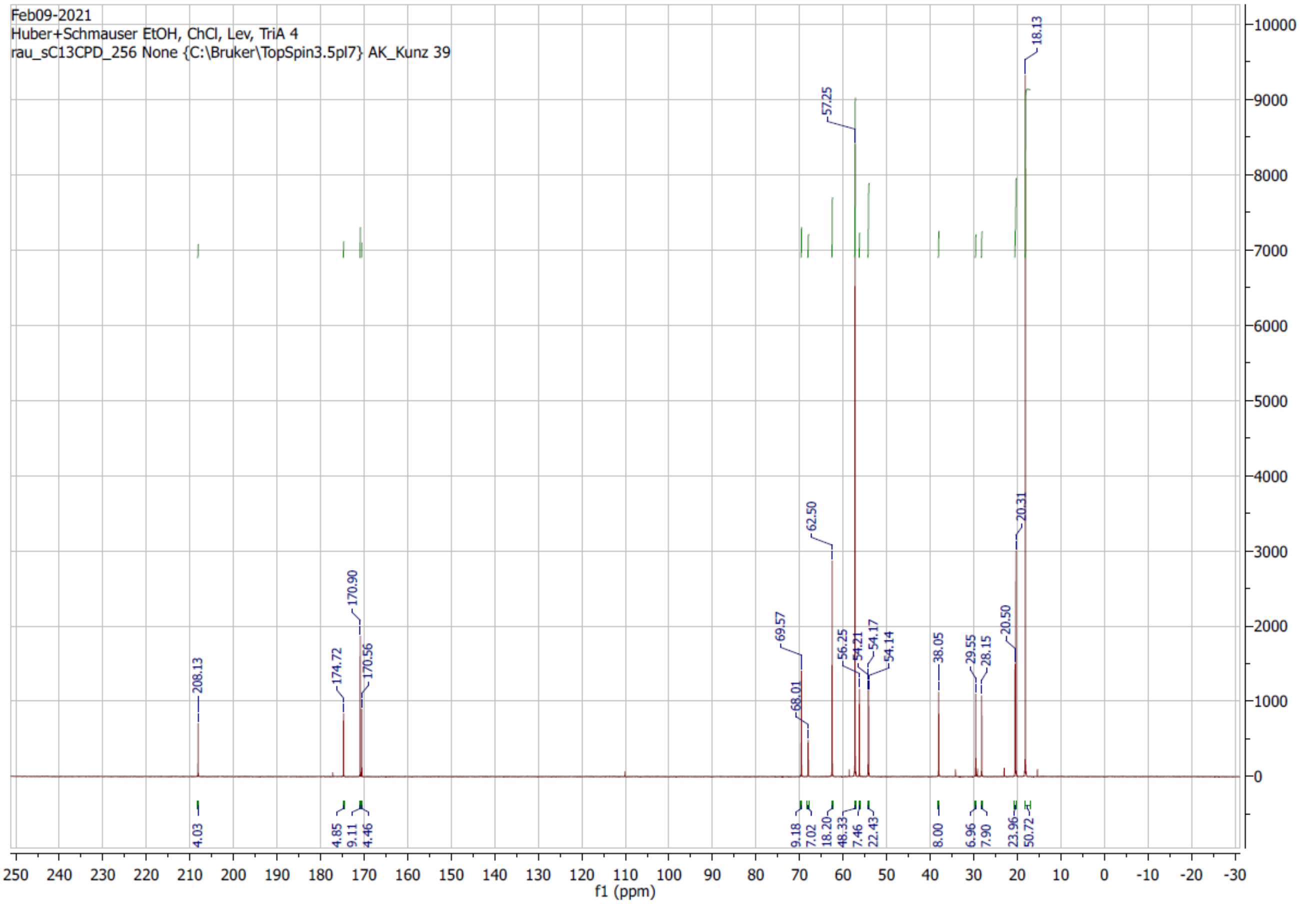

Figure S12: ^{13}C-NMR spectrum of the ternary extraction solvent of ChCl+Lev/EtOH/TriA (30:40:30) in weight three days after preparation.
